# Evaluation of chemical castration with calcium chloride versus surgical castration in donkeys: testosterone as an endpoint marker

**DOI:** 10.1186/s12917-016-0670-3

**Published:** 2016-03-08

**Authors:** Ahmed Ibrahim, Magda M. Ali, Nasser S. Abou-Khalil, Marwa F. Ali

**Affiliations:** Department of Surgery, Anesthesiology and Radiology, Faculty of veterinary medicine, Assuit University, Assuit, 70155 Egypt; Department of Medical physiology, Faculty of medicine, Assuit University, Assuit, Egypt; Department of Pathology and clinical pathology, Faculty of veterinary medicine, Assuit University, Assuit, Egypt

**Keywords:** Calcium chloride, Chemical castration, Testosterone, Donkeys

## Abstract

**Background:**

For the last few years, researchers have been interested in developing a method for chemical sterilization which may be a better alternative to surgical castration. An ideal chemical sterilant would be one that effectively arrests spermatogenesis and androgenesis as well as libido with absence of toxic or other side effects. Calcium chloride in various solutions and concentrations has been tested in many animal species, but few studies have been evaluated it in equines as a chemical sterilant. So, the objective of this study was to evaluate the clinical efficacy of chemical castration with 20 % calcium chloride dissolved in absolute ethanol in comparison with surgical castration in donkeys based on the changes in the serum testosterone level and the histopathological changes in treated testes.

**Methods:**

Twelve clinically healthy adult male donkeys were used in this study. Donkeys were divided randomly and equally into two groups: a surgical (S) group (*n* = 6) and a chemical (C) group (*n* = 6). Animals in the (S) group were subjected to surgical castration while those in the (C) group received a single bilateral intratesticular injection of 20 % calcium chloride dissolved in absolute ethanol (20 ml/testis). Animals were kept under clinical observation for 60 days. Changes in animals' behavior and gross changes in external genitalia were monitored daily. Serum concentrations of testosterone were measured prior to treatment and at 15, 30, 45 and 60 days post-treatment. Testicles in the (C) group were examined histopathologically at the end of the experiment.

**Results:**

Chemical castration with intratesticular calcium chloride vs. surgical castration failed to reduce serum concentrations of testosterone throughout the whole duration of the study; however it induced orchitis that was evident by focal necrotic areas in seminiferous tubules, cellular infiltration of neutrophils, proliferative intertubular fibrosis with a compensatory proliferation of Leydig cells. Donkeys tolerated the intratesticular injection of calcium chloride. There were no detectable changes in the general health status of the animals with the exception of swelling in external genitalia, scrotal ulcerations and fistulas. Food and water consumption and the gait of animals remained unaffected.

**Conclusion:**

Intratesticular calcium chloride can’t be considered an effective method for chemical castration in donkeys.

## Background

Castration is one of the most common equine surgical procedures and is usually performed to sterilize animals unsuitable for the genetic pool and to eliminate masculine behavior. Orchitis, epididymitis, testicular neoplasia, hydrocele, varicocele, testicular damage caused by trauma, torsion of the spermatic cord, or inguinal herniation may necessitate orchidectomy [1].

For many years, surgical castration has been considered a standard gold tool for sterilization of male animals. However, several drawbacks have been associated with this procedure such as high cost, time consumption, need for postoperative care and management, risk of post-operative complications, small-scale application, the requirement of anesthesia, medical equipment, a sterile surgical suite, a trained veterinarian, recovery time, and incision site observation [2–4].

For the last few years, researchers have been interested in developing a method for chemical castration which may be a better alternative to surgical one [2]. Different agents have been used for non-surgical chemical sterilization in domestic animals. These include intratesticular injection of cadmium chloride [5], ferric chloride and ferrous sulfate [6], glycerol [7], lactic acid [8] and calcium chloride [9]. An ideal chemical sterilizing agent would be one that effectively arrests spermatogenesis and androgenesis as well as libido with absence of toxic or other side effects [7].

Advantages of chemical castration are apparent reduction in pain and stress as well as elimination of hemorrhage, hernia, infection, myiasis and other surgical sequelae. It is also suited for mass-scale sterilization, simple and inexpensive [10].

Intratesticular injection of calcium chloride has been applied to a variety of animal species including rats [3, 4], dogs [2, 11–13], cats [14], goats [15, 16] and bulls [10, 17, 18], in a variety of formulations and concentrations. Calcium chloride has been found to cause atrophy of the seminiferous tubules and reduce circulating concentrations of testosterone and sperm counts in a dose-dependent manner in male dogs [2].

The aim of the present study was to evaluate the clinical efficacy of chemical castration with intratesticular 20 % calcium chloride dissolved in absolute ethanol in comparison with surgical castration in donkeys based on changes in circulating concentrations of testosterone and histopathological changes in treated testes. We hypothesized that calcium chloride castration would be a suitable alternative to surgical one and would possess the upper hand based on the risk/benefit analysis and welfare issue.

## Methods

### Animals

The study was carried out in accordance with the Egyptian laws and University guidelines for the animal care. All procedures of the current work have been approved by the National Ethical Committee of the Faculty of Veterinary Medicine, Assiut University, Egypt. The animals were selected in a minimum number required to obtain valid results [19].

Twelve clinically healthy, adult male donkeys, 2–3 years old and weighing 100 to 120 kg were used for this study. The animals were housed in a well-ventilated stable with water and food ad libitum.

### Experimental protocol

Donkeys were divided randomly into two equal groups: a surgical (S) group (*n* = 6) and a chemical (C) group (*n* = 6). Animals in the (S) group were subjected to surgical castration using the scrotal ablation technique while those in the (C) group received a single bilateral intratesticular injection of 20 % calcium chloride dissolved in absolute ethanol in a dose of 20 ml/testis.

### Technique of the intratesticular injection of calcium chloride solution

Under physical restraint, the animal was positioned in lateral recumbency. The scrotal area was thoroughly scrubbed. The lower testicle was injected first. The testicle was secured firmly against the skin of the scrotum. The site of injection near the tail of the epididymis was disinfected with 10 % Betadine solution. Under a complete aseptic condition, a 21G × 1.5 needle was directed from the caudoventral aspect of each testis approximately 0.5 cm from the epididymal tail towards the dorsocranial aspect of the testis. The solution was carefully deposited along the entire route by linear infiltration while withdrawing the needle from the proximal to the distal end. Necessary care was taken to prevent the seepage of solution from the injection site.

### Clinical observation

All animals were kept under clinical observation for 60 days. Changes in animals’ behavior, food and water consumption, gait of animals and gross changes in external genitalia were monitored daily.

### Blood sampling

Blood samples were collected from the jugular vein under a complete aseptic condition at 8.30 a.m. prior to and at 15, 30, 45 and 60 days from the beginning of the experiment in both groups, (S) and (C). Serum was isolated after centrifugation at 3000 rpm for 10 min stored at−20 °C until analyzed for determination of testosterone levels.

### Serum testosterone assay

It was determined by ELISA using testosterone enzyme immunoassay test kit according to the manufacturer^’^s instruction (BioCheck, Inc.) with a minimum detectable concentration of 0.05 ng/ml.

### Histopathological examination

At the end of the experiment (60 days), the testicles of donkeys in the (C) group were removed surgically under general anesthesia (intravenous thiopental, 10 mg/kg BW) [20] for histopathological examination.

Testes were sectioned transversely and examined grossly. Representative samples from gross lesions were taken. These samples were immersed in 10 % neutral buffer formalin for 2 days after removal of tunica albicans, prepared in 1 × 1 cm size and dehydrated in a graded alcohol series. Histopathological sections were prepared at a thickness of 4 μ, fixed in 10 % neutral buffered formalin, cleared with methyl benzoate then embedded in paraffin wax and cleared in methyl benzoate. These sections were stained with hematoxylin-eosin and examined by light microscope [21]. Histopathological lesions of testes were scored according to Fox et al. [22].

### Statistical analysis

The analysis was carried out using a standard statistical software program (SPSS version 16, SPSS Inc, Chicago, III). The data were expressed as means ± standard error of the mean (SEM). One-way ANOVA was used to determine the effects of each type of castration on serum testosterone levels followed by individual comparison using Tukey^’^s post-test. The effects of treatment, time, and the interaction of treatment with time were evaluated using univariate general linear model. Differences were considered statistically significant at *P* < 0.05.

## Results

### Clinical observation

**Fig. 1:**
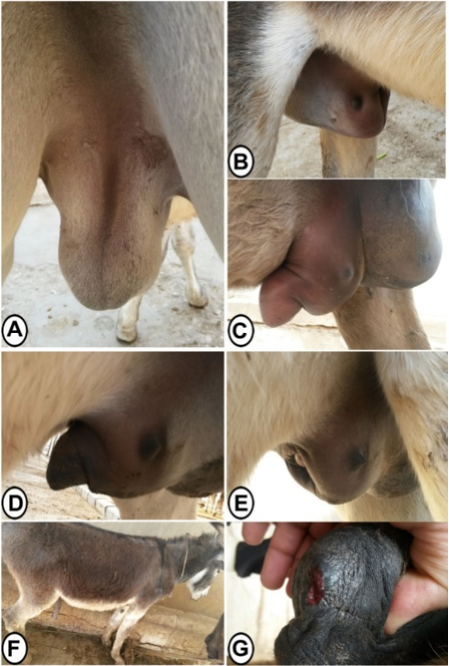
Gross changes in external genitalia in the (S) group. **a** and **b** Scrotal and preputial swelling 24 h post-intratesticular injection of calcium chloride. **c** The swelling increased severely (5^th^ day post-injection). **d** The swelling began to decrease gradually (6^th^ day post-injection). **e** and **f** Swelling subsided completely (the 14^th^ day post-injection). **g** Fistulized scrotal skin ulceration

Donkeys in the (C) group exerted mild discomfort and irritability during the intratesticular injection of 20 % calcium chloride dissolved in absolute alcohol. By the end of the injection, testes appeared to be enlarged and firm in consistency. A painful and hot swelling in the scrotum and the prepuce was evident 24 h post-injection in all donkeys (Fig. 1a and b). The swelling increased markedly from the 2^nd^ day post-injection reaching its peak at the 5^th^ day post-injection (Fig. 1c). Phimosis was recorded at the 3^rd^ day post-injection, however urination process retained unaffected. The swelling began to decay at the 6^th^ day post injection (Fig. 1d) to be subsided completely from both, the scrotum and the prepuce by the 14^th^ day post-injection (Fig. 1e and f).

Four out of six donkeys developed scrotal ulcerations and fistulas (Fig. 1g), which were managed surgically by evacuation, curetting and lavage with Betadine 10 % for several times. This was followed by daily dressing with Betadine 10 %.

There were no detectable changes in the general health status of the animals with the exception of swelling in external genitalia, scrotal ulcerations and fistulas. Food and water consumption and animal gait remained unaffected.

Donkeys in the (S) group did not record any post-surgical complications following castration by scrotal ablation technique.

### Histopathological examination

#### Gross appearance

**Fig. 2:**
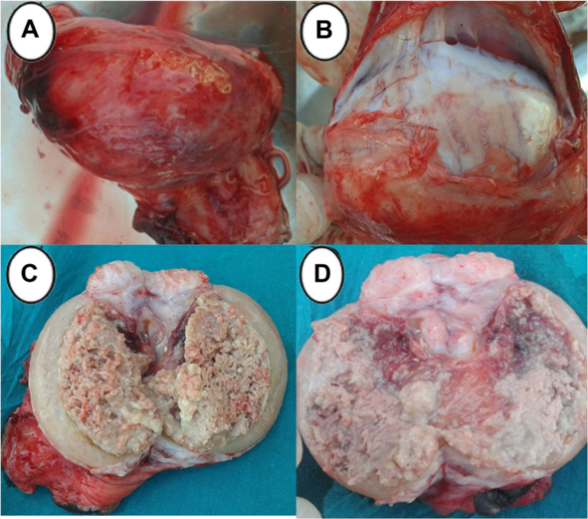
Pathological gross findings in testes in the (C) group. **a** Hemorrhagic areas on the testicular membranes. **b** Severe adhesions between tunica albuginea and tunica vaginals. **c** and **d** Testes showing necrosed and sloughed tissues in the testicular parenchyma

Grossly, the testes of donkeys in the (C) group revealed hemorrhagic areas on the parietal vaginal tunics (Fig. 2a). Orchitis presented by extensive adhesions between the visceral and parietal vaginal tunics (Fig. 2b).

**Fig. 3:**
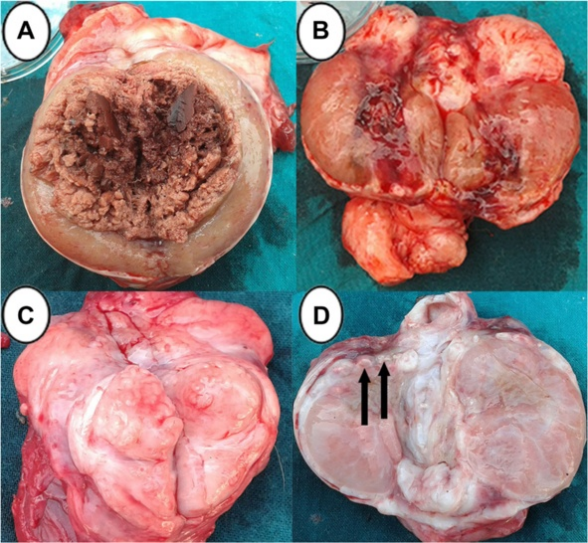
Pathological gross appearance in testes in the (C) group. **a** Hemorrhagic necrosis with dark coffee discoloration. **b** Hemorrhagic areas with softness inconsistency. **c** and **d** Atrophied testes with mineralization (arrow)

Testicular cross section showed severe necrosis and sloughing in the testicular parenchyma in two animals (Fig. 2c and d). Testes in one donkey developed hemorrhagic necrosis that manifested by dark coffee discoloration with sloughed testicular parenchyma (Fig. 3a). In another animal, testes were soft in consistency with hemorrhagic areas in the testicular parenchyma (Fig. 3b). Also, there were two animals that had atrophied testes which were firm in consistency with mineralization at the periphery of the testes (Fig. 3c and d), Table 1.

**Table 1:** Gross pathological findings in donkeys treated with calcium chloride

Gross lesions	Case number					
	1	2	3	4	5	6
Orchitis	++	+++	++	+	–	–
Necrosis & Sloghing	++++	+++	–	–	–	–
Heamorrhagic necrosis	–	–	++	++	–	–
Atrophy of testes	–	–	–	–	++	++

*The severity degree of lesions*: very severe = ++++, severe = +++, moderate = ++, mild = +, no damage = –

### Microscopic appearance

**Fig. 4:**
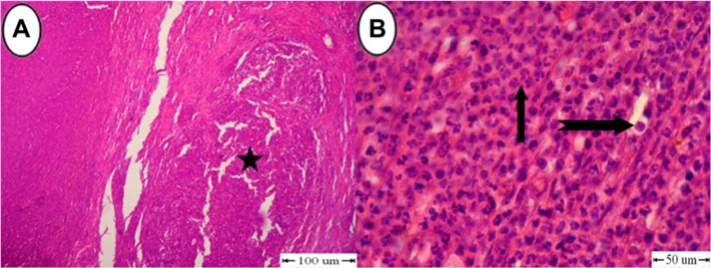
Histopathological findings in testes in the (C) group. **a** Connective tissue infiltrated with neutrophils and mononuclear cellular reactions (star). **b** High power to (Fig. 4a). Connective tissue infiltrated with a large number of neutrophils (arrow) and fewer mononuclear cellular reactions (notched arrow)

**Fig. 5:**
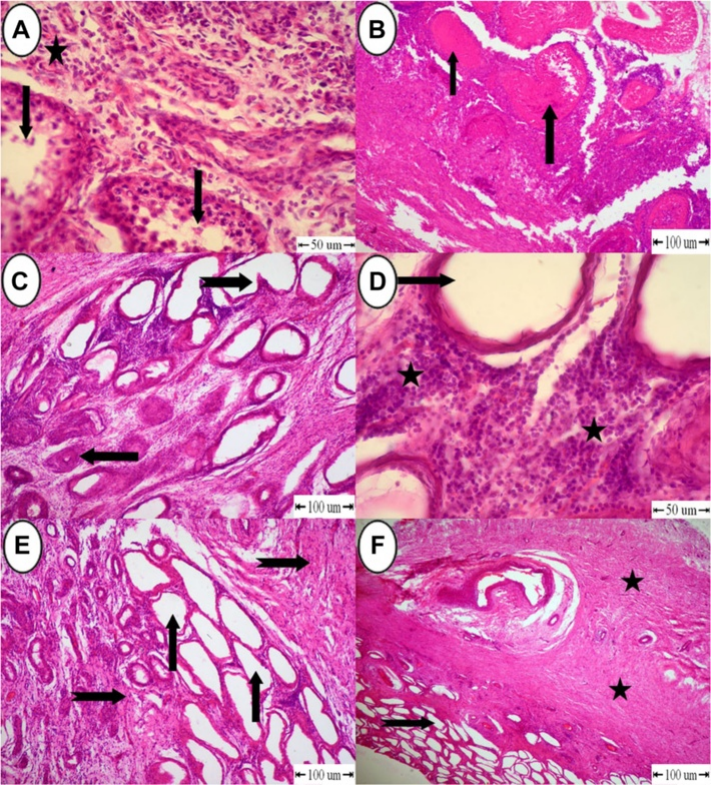
Histopathological findings in testes in the (C) group. **a** Testes showing a cross section of one seminiferous tubule with a decrease in spermatogenesis to two or three layers (arrow) and thickening of intertubular stroma (star) infiltrated with interstitial cells. **b** Testes showing coagulative necrosis of germinal epithelium and necrosed tissue collected in the lumen (arrow). **c** Testes showing empty, irregular (notched arrow) and atrophied (arrow) seminiferous tubules. **d** High power showing empty seminiferous tubules (arrow) and presence of clusters of Leydig cells. **e** Testes showing empty seminiferous tubules (arrow) and intertubular fibrosis (notched arrow). **f** Testes showing intertubular fibrosis and hyalinization (star) and presence of empty seminiferous tubules (notched arrow)

Inflammatory reaction was the main detectable finding in the histopathological examination of treated testes in the (C) group. This reaction expressed by a progressive connective tissue that was heavily infiltrated with large number of neutrophils and fewer mononuclear cellular reactions (Fig. 4a and b), Table 2. Testes from two donkeys showed a decrease in spermatogenesis which reached two or three layers of spermatogenic cells within the seminiferous tubules (Fig. 5a). Other seminiferous tubules showed diffuse tubular coagulative necrosis of germinal epithelium with necrosed tissue collected in their lumens (Fig. 5b). Most seminiferous tubules were empty, dilated with irregular basement membranes, with complete depletion of both germinal and Sertoli cells leaving only a basement membrane. Also, there were atrophied seminiferous tubules characterized by complete hyalinized scarring of the tubule with varying degrees of tubular collapse (Fig. 5c).

**Table 2:** Histopathological findings in donkeys treated with calcium chloride

Histopathological lesions	Case number					
	1	2	3	4	5	6
-Inflammatory reaction	++	++	+++	+	–	–
-Fibrosis	–	–	–	++++	+++	+++
-Decrease in layers of seminiferous tubules	–	–	–	–	+++	++
-Coagulative necrosis of germinal epithelium	–	–	−	+++	++	++++
-Empty dilated seminiferous tubules	++++	++++	+++	–	–	–
-Atrophy of seminiferous tubules	+++	++	–	–	–	–
-Heamorrhage	–	–	++	+	++	–
-Thrombosis	–	–	++	–	++	–
-Dystrophic calcification	–	–	–	–	++	+

**Fig. 6:**
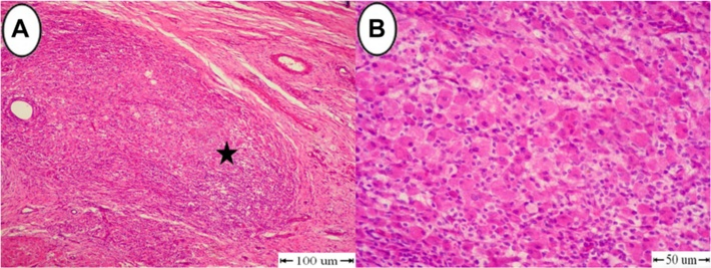
Histopathological findings in testes in the (C) group. **a** Testes showing large clusters of Leydig cells surrounded by collagen fibers (star). **b** High power showing large clusters of Leydig cells

Proliferative intertubular stroma was infiltrated with large clusters of Leydig cells. These Leydig cells were surrounded by collagen fibers (Figs. 5d, 6a and b). Hyalinization was a prominent finding in the examined cases (Fig. 5e and f).

**Fig. 7:**
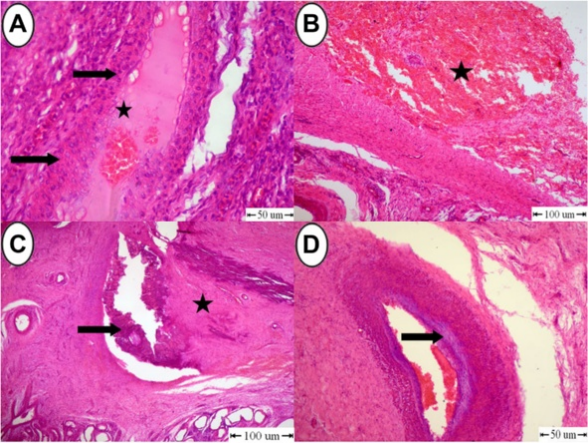
Histopathological findings in testes in the (C) group. **a** Testes showing hyperplasia of endothelial cells (arrow) and presence of a red thrombus (star). **b** Testes showing hemorrhage (star). **c** Necrosis of testicular parenchyma (star) undergoes dystrophic calcification (arrow). **d** Testes showing dystrophic calcification on the wall of blood vessels (arrow)

Vascular changes expressed by hyperplasia of endothelial cells lining blood vessels, and formation of red thrombus inside the lumen of blood vessel that consisted of RBCs and platelets (Fig. 7a and b). Dystrophic calcification was observed within large infracted areas within the testicular parenchyma and the walls of some blood vessels (Fig. 7c and d).

### Serum testosterone assay

Serum testosterone levels of surgically castrated donkeys at day 15 exhibited a significantly sudden drop (2.928 ± 0.806 ng/ml) as compared with day 0 (5.135 ± 0.574 ng/ml). Similarly at day 30, 45, and 60 it significantly maintained lower levels than day 0 (2.617 ± 0.729, 2.360 ± 0.617 and 2.889 ± 0.447 ng/ml, respectively).

**Fig. 8:**
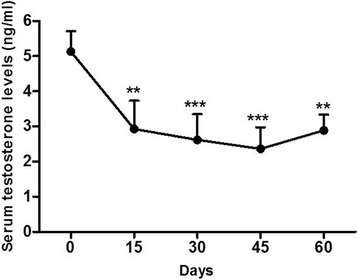
Serum concentrations of testosterone in both (S) and (C) groups. Graphic representation of changes in serum testosterone levels of donkeys at day 0 (pre-castration) vs. days 15, 30, 45, and 60 following surgical or chemical castration. Values are expressed as means ± SEM, *n* = *6* animals per group. Mean values are significantly different by repeated measures ANOVA followed by Tukey post-test. **P < 0.01 vs. day 0; ***P < 0.001 vs. day 0

On the other hand, there were no significant differences between pre-sterilization testosterone levels (5.111 ± 1.052 ng/ml) relative to post-sterilization ones at all time intervals (15, 30, 45 and 60 days) when calcium chloride was used as a chemical sterilant in the (C) group (6.153 ± 1.360, 6.033 ± 1.276, 6.847 ± 0.680 and 7.400 ± 0.406 ng/ml, respectively) Fig. 8.

## Discussion

The outcome of surgical castration risks versus its benefits necessitates a shift towards other non-invasive chemical approaches that should be characterized by full capability of arresting spermatogenesis, androgenesis and libido, effectiveness on a large scale, high safety profile, and irreversibility following single treatment. From this point of view, calcium chloride attracts much attention to be evaluated as an unconventional substitute for surgical castration in animals.

To our knowledge, the only published study which investigated chemical castration in donkeys was performed by Ali et al., [23]. They estimated the efficacy of chemical castration with intratesticular injection of calcium chloride dissolved in distilled water in concentrations of 25, 50 and 70 % by the presence or absence of sperms in testicular smears. Unfortunately, they neglected to consider its effect on serum testosterone levels, which provided the premise for this current study.

The major finding of the current study was the ineffectiveness of 20 % calcium chloride for chemical castration in donkeys. This was evident by the absence of significant changes in serum testosterone levels associated with a compensatory proliferation of Leydig cells in the histopathological sections, even so germinal epithelium and vascular endothelium are specifically targeted by calcium chloride.

We decided to apply calcium chloride in a concentration of 20 % as higher concentrations (30 % or more) were reported to have higher risk of abscessiation [18, 24], however in our study ulcerations with fistulas had been recorded between animals in the (C) group (4 donkeys). This may be attributed to absolute ethanol, as it appears to produce its effect by necrosis and infarction leading to fibrosis and sloughing of tissue [25].

In our study, the choice of absolute ethanol as a base provides the optimal formulation for calcium chloride. It has an anesthetic effect, less inflammatory reaction, fewer complications, besides high effectiveness [10, 11, 24].

Blood samples for the serum testosterone assay were taken at a fixed time at 8.30 a.m. as the highest serum testosterone levels were observed between 6.00 and 10.00 a.m. [26].

Surgical castration was supported by a significant reduction in post-castration levels of serum testosterone at all time points against pre-castration ones. This is exactly what happened in previous studies [27, 28]. This provides confirmation for the fact that testes act as a primary source of male sex hormones [29].

On the other hand, chemical castration with calcium chloride failed to induce any significant changes in serum concentrations of testosterone as approved histopathologically by the presence of proliferative Leydig cells. This was in line with the findings of Leoci [12] but contradicts others [2–4, 14, 15].

Confirming to our results, even the highest effective dose reported in literatures (60 % solution of calcium chloride) decreased testosterone levels to only near its lower limit of physiological range, and concentration up to 30 % was not enough to induce complete testicular fibrosis, whereas testosterone levels return to normal within 12-month time point by the current dose [12, 30].

Intratesticular administration of calcium chloride in donkeys produced inflammation that has been confirmed by testicular gross and histopathological changes. This inflammatory reaction was supposed to be the mechanism of calcium chloride by its irritating effect on testicular tissues [25].

The severe fibrosis in the interstitial spaces was associated with chronic inflammation. Similar findings were reported by Jana and Samanta [2]. Hyperplasia of the endothelial cells lining the blood vessels as well as thromboses of blood vessels led to ischemia resulted in swelling, firmness, and atrophy of the testicular tissues [18, 31].

Over the course of treatment, calcium chloride caused damage to the spermatogonial stem cells that may lead to filling with necrotic germ cells. Thus, it could not reinitiate spermatogenesis in these seminiferous tubules [4]. The complete hyalinized scarring of the seminiferous tubules with varying degrees of a tubular collapse was associated with inflammation induced by calcium chloride [32].

Leydig cell proliferation is likely to be a compensatory mechanism to increase testicular steroidogenesis stimulated by testosterone insufficiency [33–35]. However, our results were in disagreement with Jana and Samanta [14] who reported that there was no sign of regeneration in Leydig cells.

## Conclusion

Single bilateral intratesticular injection of 20 % calcium chloride dissolved in absolute ethanol failed to significantly reduce the serum levels of testosterone within 60 days of treatment in donkeys. It is unlikely to modify the male-like behavior of donkeys and cannot be accepted as an alternative method to the surgical castration. Further studies are warranted to explore other chemical sterilants in other solvents to achieve the highest efficacy with less adverse impacts.
