# Erratum to: Evaluation of chemical castration with calcium chloride versus surgical castration in donkeys: testosterone as an endpoint marker

**DOI:** 10.1186/s12917-016-0683-y

**Published:** 2016-03-23

**Authors:** Ahmed Ibrahim, Magda M. Ali, Nasser S. Abou-Khalil, Marwa F. Ali

**Affiliations:** Department of Surgery, Anesthesiology and Radiology, Faculty of veterinary medicine, Assuit University, Assuit, 70155 Egypt; Department of Medical physiology, Faculty of medicine, Assuit University, Assuit, Egypt; Department of Pathology and clinical pathology, Faculty of veterinary medicine, Assuit University, Assuit, Egypt

Unfortunately, after publication of this article [1] it was noticed that an incorrect version of Fig. 8 (Fig. 1 here) was introduced during the production process. The correct figure can be seen below. The original article has also been updated to reflect this.

**Fig. 1:**
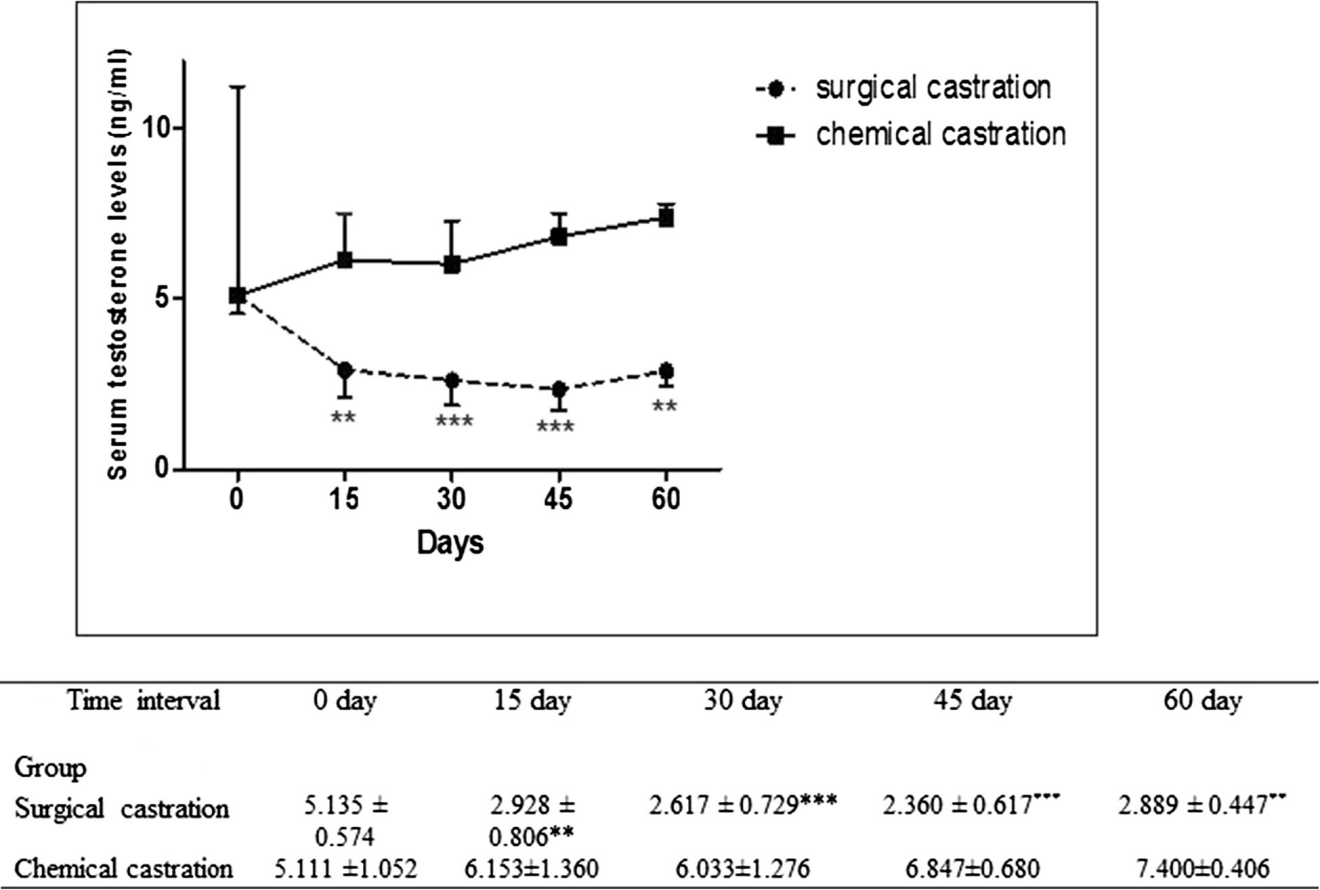
Serum concentrations of testosterone in both (S) and (C) groups. Graphic representation of changes in serum testosterone levels of donkeys at day 0 (pre-castration) vs. days 15, 30, 45, and 60 following surgical or chemical castration. Values are expressed as means ± SEM, *n* = *6* animals per group. Mean values are significantly different by repeated measures ANOVA followed by Tukey post-test. **P < 0.01 vs. day 0; ***P < 0.001 vs. day 0
